# The Domestic Grate

**Published:** 1920-07-17

**Authors:** 


**THE PUBLIC WELL-BEING—(*continued*).**

### The Domestic Grate.

### Its Injury, Waste, and Attraction.

The Ministry of Health has issued an interim report of the Departmental Committee on smoke abatement. It includes some remarkable facts on the present waste and injury to health, and the production of the report at this time is arranged with the object of getting some improvement in the construction of the many thousands of houses to which people look patiently forward. For that reason it deals with the domestic side of the question, and it will doubtless be new to many that domestic smoke effects more damage than industrial smoke.

It is stated that domestic soot contains a higher percentage of carbon and tar than factory soot, and is, therefore, more obnoxious and adhesive than the latter. At least half the total output of smoke is domestic, and at least 6 per cent. of the coal ordinarily burnt in domestic grates escapes as soot. Taking $40\frac{1}{2}$ million tons as the amount of coal burnt annually in the United Kingdom in its natural condition for domestic purposes, the loss amounts to nearly $2\frac{1}{2}$ million tons of soot (*i.e.*, waste fuel) per annum. The annual domestic consumption of crude coal in the metropolitan area is only $4\frac{3}{8}$ million tons. The report, therefore, estimates that the amount of fuel wasted throughout the country every year would warm all London for at least six months. Instead of this, it escapes into the air as soot, and befouls everything it touches.

The various aspects of the damage done by impurities in the atmosphere are briefly summarised; health, agriculture, building materials, are all most seriously affected.

#### Onslaught on "Prejudice."

There is no law forbidding the burning of raw coal—and there are a million new houses to be built in the next few years. The Committee feels strongly that the new houses must not perpetuate the smoke evil; "a unique opportunity for constructive reform" must not be lost. Accordingly the Committee has investigated various ways in which heat can be supplied to the new houses without burning raw coal in domestic grates. It makes a direct onslaught against the "prejudice" in favour of domestic grates, and while undesirous of hindering the erection of houses so urgently needed, it puts forward the drastic recommendation that sanction should be withheld from housing schemes which do not provide smokeless means of heating and cooking. The only concession to the deep-rooted "prejudice" is that in houses built with Government subsidy not more than one, or in exceptional cases two, coal grates should be installed, these to be adapted to the use of coke as well as of coal. The proposals are for the use of gas, hot-water, and electric heating; gas and electricity undertakings should be given every facility and encouragement to increase and cheapen supplies; and municipal undertakings should not overcharge in order to relieve the rates. Attention is drawn to a central boiler installation, which is to be used in a colony of 2,000 houses to be built for the Manchester Corporation. The water will be laid on to the sink and to the bath, and a constant supply at 150° Fah. will be available day and night. In this way during the summer the house will be quite smokeless (cooking being done by gas), and at the same time the housewife will have the advantage of a constant supply of hot water and of a very considerable saving of labour in carrying coals and cleaning. The service will be better and the labour less, while all the fuel burnt is concentrated in one furnace under a skilled stoker. When a central supply is not practicable, the cheapest and most efficient way of providing hot water is by a coke-fired boiler.

#### Admirable but Unattractive.

This all sounds admirable, and it would no doubt be a cleaner and healthier land to live in if the

**THE PUBLIC WELL-BEING**—(*continued*).

proposals could be put into force, and the existing domestic arrangements gradually eliminated. But the Committee realises the universal affection for open grates, and in its concession of at least one in a house it must recognise that the vast majority of householders find one grate is quite sufficiently expensive at present prices, so there would not be much ultimate improvement. It would be interesting, by the way, to know if any difference is noticeable in air pollution by the necessarily greater economy in the use of coal as compared with the pre-war period. At the same time the Committee's general proposals are sound, and we should have more comfortable and equable—if not such attractive—heating if we carried them out. But who would not rather huddle over a red-hot fire and dream dreams than try to seek inspiration from the dull monotony of water-pipes for the mere satisfaction of equable warmth and a layer or two less of filth on the Houses of Parliament? It was stated at a meeting of the Institute of Sanitary Engineers that the Americans solve this problem, when they can afford it, by burning wood logs on dogs in an open hearth.

